# Will Transcranial Magnetic Stimulation Improve the Treatment of Obsessive–Compulsive Disorder? A Systematic Review and Meta-Analysis of Current Targets and Clinical Evidence

**DOI:** 10.3390/life13071494

**Published:** 2023-07-01

**Authors:** Giacomo Grassi, Corinna Moradei, Chiara Cecchelli

**Affiliations:** Brain Center Firenze, 50144 Florence, Italy

**Keywords:** TMS, OCD, SMA, DLPFC, ACC, mPFC, OFC, deepTMS, iTBS, cTBS

## Abstract

Background: Although in 2017 a repetitive transcranial magnetic stimulation (rTMS) protocol received Food and Drug Administration approval for the first time for the treatment of obsessive–compulsive disorder (OCD), which neural target and which protocol should be used for OCD are still debated. The aim of the present study was to perform a systematic review and meta-analysis of the available open and sham-controlled trials. Methods: The primary analysis included a pairwise meta-analysis (over 31 trials), and then subgroup analyses were performed for each targeted brain area. Meta-regression analyses explored the possible moderators of effect size. Results: The pairwise meta-analysis showed a significant reduction in OCD symptoms following active rTMS (g = −0.45 [95%CI: −0.62, −0.29]) with moderate heterogeneity (I^2^ = 34.9%). Subgroup analyses showed a significant effect of rTMS over the bilateral pre-SMA (supplementary motor area), the DLPFC (dorsolateral prefrontal cortex), the ACC/mPFC (anterior cingulate cortex and medial prefrontal cortex), and the OFC (orbitofrontal cortex). No moderators of the effect size emerged. Conclusions: TMS of several brain targets represents a safe and effective treatment option for OCD patients. Further studies are needed to help clinicians to individualize TMS protocols and targets for each patient.

## 1. Introduction

Obsessive–compulsive disorder (OCD) is a chronic condition affecting 1–3% of the general population, characterized by repeated experience of unwanted, distressing, and intrusive thoughts (obsessions) and/or repetitive or ritualized behaviors (compulsions), usually performed to reduce obsession-associated anxiety. Selective serotonin reuptake inhibitors (SSRIs) and exposure and response prevention (ERP)-based cognitive–behavioral therapy (CBT) are two strongly evidence-based first-line approaches, well established in clinical practice internationally and summarized in consensus guidelines [1,2]. However, up to 40–60% of patients still do not respond to these treatments [3]. Consequently, in the last 20 years, many efforts have been made in order to find effective augmentation strategies for SSRIs and/or CBT. In OCD pharmacology, a number of options for treatment-resistant patients now exist. However, after SSRIs, no other pharmacological agents have been approved for OCD by the principal regulatory agencies (such as the Food and Drug Administration (FDA) in the United States). On the other hand, neuromodulation techniques have become more and more relevant for the treatment of several psychiatric disorders and both the invasive technique of deep brain stimulation (DBS) and the non-invasive technique of transcranial magnetic stimulation (TMS) have received FDA approval for the treatment of OCD. Indeed, DBS of the anterior limb of the internal capsule was granted humanitarian device exemption in 2009 by the FDA for the treatment of severely debilitating, treatment-refractory OCD, while deep TMS of the anterior cingulate cortex and dorsomedial prefrontal cortex received FDA approval for OCD in 2017 [4]. However, beyond FDA approval for the deep TMS protocol, several other TMS protocols over different neural targets have been studied in the last 15 years and there is still not a clear consensus on which protocol should be recommended in clinical practice. Thus, the aim of the present paper was to conduct a systematic review and a meta-analysis of existing studies on TMS for OCD in order to answer a series of relevant clinical questions: (1) is TMS effective for the treatment of OCD? (2) Is there a neural target and/or TMS protocol that seem to be better than others? (3) Is there a profile of the optimal OCD candidate for TMS treatment?

## 2. Materials and Methods

This report was written according to the Preferred Reporting Items for Systematic Reviews and Meta-Analyses (PRISMA) guidelines [5]. PRIMA checklist is available in Supplementary Materials.

### 2.1. Search Strategy and Eligibility Criteria

A comprehensive search of the literature was performed by consulting four databases: PubMed, APA PsychInfo, Web of Science, and Cochrane Library from the earliest publication up to December 2022. The search keywords used were (“obsessive compulsive disorder” OR “OCD” or “obsessions” OR “compulsions”) AND (“magnetic stimulation” OR “rTMS” OR “transcranial magnetic” OR “theta burst stimulation” OR “iTBS” OR “cTBS” OR “TMS” OR “dTMS”). Two authors (G.G. and C.M.) independently performed the literature search. 

The following studies were included in the systematic review: (1) open trials, randomized controlled trials (RCTs), and single- or double-blinded trials with either a parallel or crossover design enrolling adult patients (≥18 years) with a primary diagnosis of OCD according to the DSM-5 (APA, 2013) or the ICD-11; (2) studies including the Yale–Brown Obsessive–Compulsive Scale (Y-BOCS) assessment to evaluate OCD symptom changes before and after treatment [6]; (3) trials using any form of rTMS stimulation (i.e., rTMS, iTBS, cTBS, dTMS, alpha-guided rTMS, with or without neuronavigation) and/or sham TMS (i.e., sham coil, tilted coil, or deactivated coil) for at least five treatment sessions with or without adjuvant/add-on treatment; (4) studies with at least five subjects per branch of treatment (active or sham); and (5) peer-reviewed and English-language studies. The following studies were excluded from the systematic review: (1) review articles, single-case studies, case series, case reports, and retrospective studies; (2) studies on subjects with OCD not as the primary diagnosis; (3) trials with fewer than five treatment sessions and/or fewer than five subjects and/or not using the Y-BOCS assessment; and (3) studies using non-repetitive TMS such as single- or double-pulse TMS. The meta-analysis was conducted on the studies fulfilling the inclusion and exclusion criteria but was limited to sham-controlled trials.

### 2.2. Study Selection

The first step of the analysis focused on eliminating duplicate studies. Then, each title of the selected studies was reviewed excluding those that were ineligible. As a third step, the abstracts were evaluated removing those that did not meet the inclusion/exclusion criteria. Finally, the remaining studies had their full texts reviewed. Data extraction was conducted independently by two researchers (G.G. and C.C.). In the event of any disagreement, the research group took part in discussions in order to reach consensus.

### 2.3. Data Extraction and Outcome Measures

Data meeting the inclusion criteria were extracted and placed in a structured Excel sheet by C.M. The extraction of the study data into tabulated spreadsheets was carried out by G.G. and double-checked by C.C. The following data were researched from each study: (1) treatment effects: pre- and post-treatment YBOCS scores; (2) treatment protocol: coil type, TMS frequency and intensity of stimulation, stimulation location, method of identification of stimulation location, number of pulses per session and total pulses, total number of sessions, type of sham condition, and symptom provocation during TMS treatment; (3) tolerability and safety: dropout rates and presence of serious adverse events (AE); and (4) other potential moderator variables: mean age, sex distribution, ongoing treatments (medication or psychotherapy), rates of comorbidities, and treatment resistance. When means and standard deviations were not available, these were calculated from the available data, following guidance in the Cochrane Handbook [7]. When standard deviations could not be calculated from the available data, they were imputed according to Wan et al [8]. The study authors were contacted in case of missing data for the primary outcome measure. In case of no response, the missing data were extracted from other studies, but when data were unavailable, the study was excluded from the meta-analysis.

### 2.4. Risk of Bias Assessment

The risk of bias was calculated according to the Cochrane risk of bias tool and each study was classified as high risk, unclear risk, or low risk [9].

### 2.5. Statistical Analysis

Pairwise meta-analyses were carried out using the Statistical Package for the Social Sciences v29 (SPSS). Hedge’s g based on the random effects model was used to estimate the effect of the active rTMS group versus the sham rTMS control group. Following Cohen’s convention, an effect size of 0.2 was considered small, 0.5 was considered moderate, and 0.8 was considered large. The effect size was calculated by reporting the sample size, mean, and standard deviation of the Y-BOCS post-treatment scores of the active and sham groups for each study. The post-treatment score was favored over multiple follow-ups, with it being the most frequent measure reported across all papers. For crossover trials, only the measures before the crossover were considered. In the case of studies with more than one type of rTMS, matched with sham, the sham’s sample size was split following the recommendation of the Cochrane Handbook [10].

Subgroup analyses of the primary pairwise meta-analysis were carried out. Hedge’s g based on random or fixed effects models were selected depending on the homogeneity test. The subgroups were based on: (1) brain target; (2) rTMS vs. cTBS over the pre-SMA (3) stimulation of different brain sides over the DLPFC; and (4) high- or low-frequency stimulation for each side of the brain and targeted area. A meta-regression analysis was also carried out to explore the possible effect of continuous and categorical moderator variables of the rTMS effect (total number of stimuli, total number of sessions, mean age of the participants, and percentage of females in the active group). Heterogeneity was assessed using the I^2^ statistic and interpreted following the Cochrane guidelines [7]. Publication bias was evaluated by checking funnel plots to test for any asymmetry. Moreover, Egger’s regression test [11] was used to infer a possible problem of publication bias.

## 3. Results

### 3.1. Systematic Review and Meta-Analytic Results Overview 

**Figure 1:**
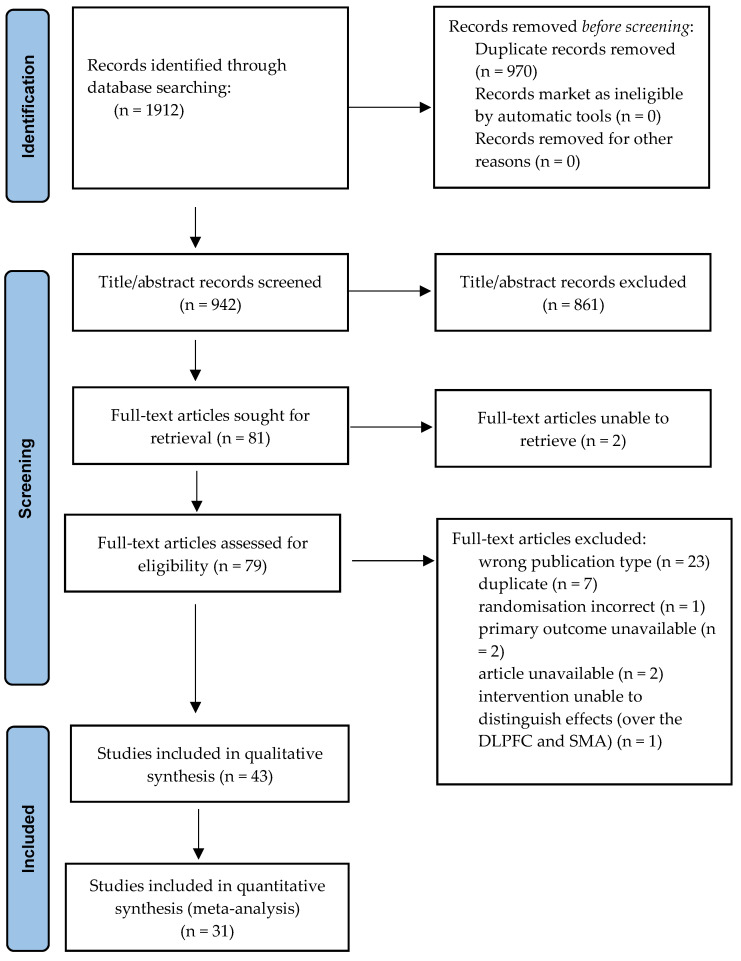
Flow diagram illustrating the selection of the studies for inclusion in the systematic review and meta-analysis.

Forty-three studies of the 1912 initial records were selected and included in the systematic review, and 31 of these were included in the meta-analysis (see Table 1 and Figure 1) [12,13,14,15,16,17,18,19,20,21,22,23,24,25,26,27,28,29,30,31,32,33,34,35,36,37,38,39,40,41,42,43,44,45,46,47,48,49,50,51,52,53,54]. The studies were grouped according to the brain area targeted with TMS. For the meta-analysis, we excluded two studies [34,37] because of missing data that could not be obtained (Y-BOCS means and standard deviations pre and/or post treatment). Two other studies were excluded from the analyses due to publication bias [14,29]. A total of 485 patients with active TMS and 407 patients with sham rTMS were evaluated. The study by Kang et al. [53] was not included given that the stimulation protocol of this study sequentially targeted two brain areas (the right DLPFC and the pre-SMA). The primary analysis showed a significant reduction in the post-treatment Y-BOCS score in the active TMS group compared to the sham TMS group (Hedge’s g = −0.45 [95%CI: −0.62, −0.29], *p* < 0.001). The heterogeneity was moderate (Q(df = 29) = 45.88, I^2^ = 34.9%, *p* = 0.024). Egger’s regression intercept was not significant (intercept = 0.35, *p* = 0.34). The risk of bias calculated according to the Cochrane risk of bias tool revealed a generally reasonable quality, with most studies receiving a low or unknown risk of bias score. Egger’s regression test did not show problems of publication bias.

### 3.2. Clinical Moderators of Post-Treatment Y-BOCS Score Reduction 

A meta-regression of the initial pairwise meta-analysis was carried out in order to verify the possible effect of different moderators in decreasing Y-BOCS scores for the active group. The moderators considered were age, the percentage of females, the total number of sessions, the total number of stimuli, and the frequency of stimulation. No differences in the TMS effect were found with these types of moderators (F = 4.71, *p* = 0.58).

### 3.3. Systematic Review and Meta-Analytic Results Grouped for Brain Targets

The rationale for TMS across different brain targets in OCD is based on its mechanism of action. Indeed, TMS is able to modulate neural networks by inducing an excitatory effect (through high-frequency rTMS or intermittent theta burst (iTBS) protocols) or an inhibitory effect (through low-frequency rTMS or continuous theta burst (cTBS) protocols) on the targeted area. In addition, TMS is known to induce long-lasting changes in neural networks through long-term potentiation and long-term depression mechanisms and by inducing neurotrophic factors increase in the brain (e.g., BDNF). Therefore, TMS has been hypothesized to induce acute and long-lasting changes in the cortico-striatal loops implicated in OCD pathophysiology. Here, we summarized the rationale and the current evidence of TMS for OCD for each brain target from a Research Domain Criteria perspective. 

#### 3.3.1. Pre-Supplementary Motor Area (pre-SMA) Studies

##### Systematic Review Results

From the Research Domain Criteria (RdoC) perspective, the pre-supplementary motor area (pre-SMA) is a relevant node of the brain network involved in action planning, selection, initiation, and termination (www.nimh.nih.gov (accessed on 10 March 2023)). For this role, it is involved in the neurobiology of both impulsivity and compulsivity. Several studies have shown its involvement in OCD and especially in the impaired motor response inhibition observed in OCD patients [55,56]. Thus, the pre-SMA became a TMS target more than 10 years ago (with the first study by Mantovani et al. in 2010 [12]), and it has been largely investigated in the last few years. According to a recent international survey, the pre-SMA is the most used TMS target across specialized OCD centers providing neuromodulation for their patients (around 48% of centers) [57]. In our systematic review, we found nine double-blind sham-controlled (DBPC) trials and four open-label studies (see Table 1). Of the thirteen studies, only three are multi-center studies (two in the same country and one across two countries). In most studies, the patients were treatment resistant and under current medication (one controlled and one open trial were on non-resistant patients; in one controlled study, the patients started medication together with TMS; one open trial did not report the medication status). Comorbid patients were excluded in only one controlled study. Apparently, none of the studies were sponsored by a TMS manufacturer. Both the sham-controlled and the open trials have small sample sizes (ranging from 9 to 25 patients for the active branch in the controlled trials and from 8 to 35 patients in the open trials). All studies used a figure-8-shaped coil (of different brands). Of the sham-controlled trials, seven out of nine used a sham coil while two trials used a tilted coil as a sham procedure. The pre-SMA was detected with the 10–20 EEG system in 10 out of 12 studies while 2 studies used a neuronavigation system. Ten studies used a 1 Hz rTMS protocol while three studies used a cTBS (continuous theta burst stimulation) protocol. The number of sessions ranged from 10 to 30 and the total amount of stimuli ranged from 12,000 to 30,000. None of the studies used symptom provocation during the TMS sessions. Only six studies reported a follow-up assessment (ranging from 8 to 12 weeks). No serious side effects occurred across all studies and mild and transient headache was the most frequently reported side effect.

##### Meta-Analytic Results 

**Figure 2:**
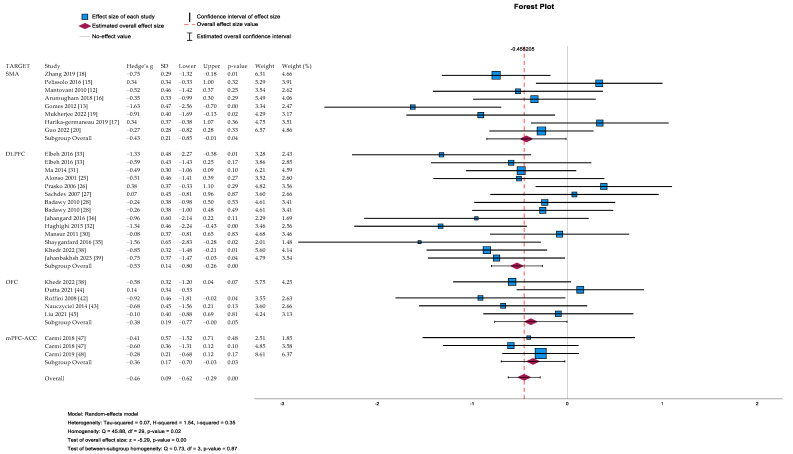
Pairwise meta-analysis forest plot: Random effects meta-analysis of Y-BOCS following TMS for OCD for active vs. sham TMS. SMA = supplementary motor area; DLPFC = dorsolateral pre-frontal cortex; OFC = orbitofrontal cortex; mPFC-ACC = medial pre-frontal cortex/anterior cingulate cortex [12,13,15,16,17,18,19,20,25,26,27,28,30,31,32,33,35,36,38,39,42,43,44,45,47,48].

Eight out of nine of the DBPC studies were included in the meta-analysis. Hawken et al. [14] were excluded because of publication bias. The effect of TMS treatment delivered over the bilateral pre-SMA revealed a significant reduction in the post-treatment Y-BOCS score (Hedge’s g = −0.43 [95%CI: −0.85, −0.014], *p* = 0.043) with a moderate effect size and high heterogeneity (I^2^ = 64%). Egger’s regression intercept was not significant (intercept = 0.98, *p* = 0.44). Possible differences in effect sizes were investigated for different TMS protocols (rTMS vs. cTBS) over the bilateral pre-SMA. The effect of both rTMS and cTBS was not significant in changing the post-treatment Y-BOCS score in the active group compared to the sham group (Hedge’s g = −0.54 [95%CI: −1.13, 0.50], *p* = 0.07, I^2^ = 70% vs. Hedge’s g = −0.27 [95%CI: −0.93, 0.39], *p* = 0.42, I^2^ = 64%) (see Figure 2). 

#### 3.3.2. Dorsolateral Prefrontal Cortex (DLPFC) Studies

##### Systematic Review Results

From the RDoC perspective, the dorsolateral prefrontal cortex (DLPFC) is a relevant node of the brain network involved in cognitive control (goal selection and response selection/inhibition) and working memory (flexible updating, interference control, and limited capacity) (www.nimh.nih.gov (accessed on 10 March 2023)). The DLPFC has been targeted in TMS trials for its role in cognitive control and for its putative indirect control of other brain areas implicated in OCD neurobiology (such as the anterior cingulate cortex or the orbitofrontal cortex) [58]. According to a recent international survey, the DLPFC represents a TMS target in 22% of specialized OCD centers providing neuromodulation for their patients [57]. In our systematic review, we found 15 sham-controlled trials (11 double-blind sham-controlled, 1 single-blind sham-controlled, and 3 cross-over sham-controlled) and 2 open-label studies. None of the 17 studies are multi-center studies. Twelve out of seventeen studies were on resistant patients and in all studies, the patients were taking medication on an ongoing basis (except for one branch of a single study that included unmedicated patients). Comorbid patients were clearly excluded in three studies and clearly included in four studies (all the other studies did not clearly report the percentage of comorbid patients). Apparently, none of the studies were sponsored by a TMS manufacturer. Both the sham-controlled and the open trials have small sample sizes (ranging from 5 to 25 patients for the active branch in the controlled trials and 7 to 27 patients in the open trials). All studies used a figure-8-shaped coil (of different brands) (except one study not reporting the type of coil used). Of the sham-controlled trials, 10 out of 15 used a tilted coil as a sham procedure, 3 used a deactivated coil, and only 2 used a proper sham coil. The DLPFC was detected with the 10–20 EEG system in 15 out of 16 studies while only 1 study used a neuronavigation system. Seven studies targeted the right DLPFC, 6 studies targeted the left DLPFC, and 4 studies targeted the DLPFC bilaterally. Seven studies used a 1 Hz rTMS protocol, 4 studies used a 10 Hz protocol, 4 studies used a 20 Hz protocol, 1 study used an alpha-guided protocol (frequency ranging from 8 to 12 Hz according to EEG data), 1 study used an intermittent TBS (iTBS) protocol, and 1 study used a continuous TBS (cTBS) protocol. Across different studies, the right and left DLPFC were targeted with both high- and low-frequency protocols. The number of sessions ranged from 10 to 30 and the total amount of stimuli ranged greatly from 15,000 to 90,000. None of the studies used symptom provocation during the TMS sessions. Eleven out of 17 studies reported a follow-up assessment (ranging from 1 to 36 weeks). No serious side effects occurred across all studies and mild, and transient headache was the most frequently reported side effect.

##### Meta-Analytic Results 

Twelve out of seventeen studies were included in the meta-analysis. The active branches of two studies with three different branches (two active branches stimulating the DLPFC at different frequencies and one sham branch) were considered separately by splitting the sham control group as explained in the methods [28,33]. The analysis revealed a significant moderate effect of rTMS treatment in reducing the Y-BOCS post-treatment score for the active group (Hedge’s g = −0.53 [95%CI: −0.80, −0.26], *p* < 0.001, with moderate heterogeneity (I = 35.2%), Egger’s intercept = 0.60, *p* = 0.92). Differences in treatment efficacy were evaluated for different stimulation protocols over the DLPFC. Differences in effect sizes were evaluated for stimulation over the left DLPFC vs. bilateral DLPFC vs. right DLPFC. The analyses revealed that the stimulation of the rDLPFC and bilateral DLPFC showed a significant decrease in the post-treatment Y-BOCS score for the active group (Hedge’s g = −0.17 [95%CI: −0.51, 1.66], *p* = 0.32, I^2^ = 22% vs. Hedge’s g = −0.85 [95%CI: −1.27, −0.42] *p* < 0.001, I^2^ = 21.5% vs. Hedge’s g = −0.64 [95%CI: −0.99, −0.29], *p* < 0.001, I^2^ = 16%). In particular, the stimulation of both sides of the DLPFC seems to produce a greater decrease in the Y-BOCS score compared to the right DLPFC (g = −0.85 vs. g = −0.64). Subsequently, differences in the treatment effect were investigated within studies where rTMS was delivered over the left DLPFC with either high or low frequency. No significant effects were found (Hedge’s g = −0.33 [95%CI: −0.71, 0.53], *p* = 0.09 vs. Hedge’s g = −0.38 [95%CI: −0.33, 1.10], *p* = 0.29). A similar comparison (high frequency versus low frequency) was also carried out for rTMS stimulation on the right DLPFC. A significant effect size was found for stimulation with low frequency on the right DLPFC (Hedge’s g = −0.30 [95%CI: −0.85, 0.25], *p* = 0.29, I^2^ = 0% vs. Hedge’s g = −0.87 [95%CI: −1.33, −0.42], *p* < 0.001, I^2^ = 0%) (see Figure 2).

#### 3.3.3. Orbitofrontal Cortex (OFC) Studies

##### Systematic Review Results

The orbitofrontal cortex (OFC) has been consistently involved in OCD pathophysiology since early neuroimaging studies more than 20 years ago [59]. Since then, many neuroimaging studies have described OFC hyperactivity in OCD patients vs. controls. Thus, the OFC represents a core node, the so-called cortico-striato-thalamo-cortical (CSTC) loop involved in OCD pathophysiology [60]. This fact has also been corroborated in an influential preclinical study using optogenetic techniques [61]. Indeed, this study showed that optogenetic-induced OFC-ventral striatum hyperactivity is able to generate compulsive-like behaviors in mice that are reversed by chronic fluoxetine administration. From the RDoC perspective, the OFC is a relevant node of the brain networks involved in both positive valence system (reward responsiveness: processes evoked by the initial presentation of a positive reinforcer as reflected by indices of neuronal activity and verbal or behavioral responses) and negative valence system (loss: a state of deprivation of a motivationally significant con-specific, object, or situation) (www.nimh.nih.gov (accessed on 10 March 2023)). According to a recent international survey, the OFC represents a TMS target in 14.8% of specialized OCD centers providing neuromodulation for their patients [57]. In our systematic review, we found five sham-controlled trials (four double-blind sham-controlled and one cross-over sham-controlled). None of the five studies were multi-center studies. Four out of five studies were on resistant patients and in all studies, the patients were taking medication on an ongoing basis. All the studies did not clearly report the rate of comorbid patients. Apparently, none of the studies were sponsored by a TMS manufacturer. All the OFC studies have small sample sizes (ranging from 10 to 20 patients for the active branch). Three studies used a figure-8-shaped coil (of different brands). Three studies used a sham coil while the other two used a tilted coil as a sham procedure. The OFC was detected with the 10–20 EEG system in all studies. Three studies targeted the right OFC while the other two targeted the left OFC. Three studies used a 1 Hz rTMS protocol and two studies used a continuous TBS (cTBS) protocol. The number of sessions ranged from 10 to 20 and the total amount of stimuli ranged greatly from 6000 to 15,000. None of the studies used symptom provocation during the TMS sessions. Four of the five studies reported a follow-up assessment (ranging from 2 to 12 weeks). No serious side effects occurred across all studies, and mild and transient headache was the most frequently reported side effect.

##### Meta-Analytic Results 

A meta-analysis carried out on five studies showed that TMS delivered on the OFC provided a significant reduction in the post-treatment Y-BOCS score in the active group compared to the sham group (Hedge’s g = −0.39 [95%CI: −0.76, −0.004], *p* = 0.048, I^2^ = 21%, Egger’s intercept = 0.80, *p* = 0.62). Subsequently, differences in treatment effects were investigated within studies where TMS was delivered either over the left OFC or the right OFC. The results revealed a significant effect size for TMS over the right OFC (Hedge’s g = −0.46 [95%CI: −0.89, −0.035], *p* = 0.034, I^2^ = 0%), whereas no significant effect was found for TMS over the left OFC (Hedge’s g = −0.24 [95%CI: −0.77, 0.29], *p* = 0.37, I^2^ = 70%) (see Figure 2). 

#### 3.3.4. Medial Prefrontal Cortex (mPFC)/Anterior Cingulate Cortex (ACC) Studies

##### Systematic Review Results

The medial prefrontal cortex (mPFC)/anterior cingulate cortex (ACC) have been the most recently targeted circuitries in TSM studies for OCD. This target has been achieved through the use of a deep TMS coil (the so-called H7-coil, manufactured by Brainsway), and the protocol from the Carmi et al. 2019 [48] study became the first TMS protocol approved by the FDA for the treatment of OCD (for more details see the above study). Recently, the FDA approved another coil (the DB-80 coil manufactured by Magventure) for the treatment of OCD, despite some authors arguing against this approval, suggesting that the two coils (the H7 and the DB-80) create different current flow and stimulated areas [62]. The two targets (mPFC and the ACC) are targeted together and simultaneously since the available coils reach the ACC, passing through (and therefore stimulating) the dorsal mPFC. The anterior cingulate cortex (ACC) is a spot of the hyperactive cortico-striatal cortical-thalamic (CSCT) loop described in OCD [59]. In addition, the ACC has consistently been reported to be involved in processes that are impaired in OCD, including the integration of thought, motivation, and emotion with movement, response selection before a movement occurs, error monitoring, and the detection of cognitive conflicts [63,64,65,66]. From the RDoC perspective, the ACC is a relevant node of several brain networks involved in different systems. In the positive valence system, the ACC is involved in the reward responsiveness and reward valuation construct; in the negative valence system, it is involved in the acute threat construct (specifically fear); in the cognitive system, it is involved in the performance monitoring subconstruct; and in the social communication system, it is involved in the reception of facial communication (www.nimh.nih.gov (accessed on 10 March 2023)). According to a recent international survey, the ACC represents a TMS target in 3.7% of specialized OCD centers providing neuromodulation for their patients [57]. From the RDoC perspective, the mPFC is a relevant node of the brain networks involved in both the positive valence system (reward learning and specifically the sub-construct habit: sequential, repetitive, motor, or cognitive behaviors elicited by external or internal triggers that, once initiated, can go to completion without constant conscious oversight) and the sensory–motor system (inhibition and termination: processes involved in the inhibition of motor plans, either before or after an action is initiated, and the sense that a motor plan has been successfully completed.) (www.nimh.nih.gov (accessed on 10 March 2023)). All these networks have been potentially implicated in OCD neuroimaging studies and are thought to be substantially implicated in OCD pathophysiology. In our systematic review, we found two sham-controlled trials and four open-label studies. One of the two sham-controlled studies was a multi-center study (the [48] Carmi et al. 2019 study that achieved FDA approval), and one of the four open-label studies was a multi-center study. Three out of six studies were on resistant patients (in the other three, studies the resistance status was not specified) and in all studies, the patients were taking medication on an ongoing basis. Comorbid patients were excluded in the two controlled trials. The two sham-controlled studies and one open multi-center study were sponsored by a TMS manufacturer (Brainsaway). The sample size across the studies ranged from 8 to 42 patients for the active branch of the sham-controlled studies and ranged from 15 to 185 in the open-label studies. All studies except one (using the DB-80 Magventure coil) used the H7 Brainsway coil. The two controlled studies used a sham coil as a sham procedure. The mPFC/ACC was detected with the 10–20 EEG system in all studies. Except for a branch of a controlled trial using a 1 Hz rTMS protocol, all the other studies used a 20 Hz protocol. The number of sessions ranged from 25 to 30 and the total amount of stimuli ranged greatly from 20,000 to 60,000. All the studies used symptom provocation during the TMS sessions. The two controlled studies reported a follow-up assessment of 4 weeks. Only one serious side effect occurred across all studies (one patient with suicidal ideation that required hospitalization). Of note, a single open-label study targeted the bilateral dorsomedial prefrontal cortex (dmPFC) with a 10 Hz protocol (6000 pulses per session) (using the DB-80 Magventure coil) and found a bimodal distribution of rTMS response [46].

##### Meta-Analytic Results 

Two studies were included in the meta-analysis; one of these had two different branches [47] (each one stimulating the mPFC/ACC at different frequencies), which were considered with both of them splitting the sham control group as explained in the methods. For the post-treatment Y-BOCS score, Hedge’s g = −0.36 [95%CI: −0.70, −0.027], *p* = 0.034 (with heterogeneity (I^2^ = 0%), Egger’s intercept = −0.16, *p* = 0.78), indicating small but significant improvement in Y-BOCS in the active group compared to the sham group (see Figure 2). 

#### 3.3.5. Multi-Target Studies

Only a few studies to date have tried to target more than one target sequentially. One study described a case series of patients targeted sequentially over the left DLPFC with either iTBS or 10 Hz protocols followed by 1 Hz stimulation of the bilateral pre-SMA and of the right OFC (this latest area was targeted only for those subjects still not responding to the DLPFC-SMA protocol after 10 sessions) [54]. On the other hand, a controlled study on 20 patients (10 for the branch) found no significant effects with respect to a sham treatment of a sequential 1 Hz stimulation of the right DLPFC and of the bilateral pre-SMA [53].

## 4. Discussion

Although only TMS over the mPFC/ACC has received FDA approval for OCD, several TMS protocols over different brain targets seem to be effective and safe for OCD patients. The meta-analytic results of this study showed that TMS over the bilateral pre-SMA, the DLPFC (especially the bilateral DLPFC), the OFC (especially the right OFC), and the mPFC/ACC seems to be more effective than sham stimulation in reducing OCD symptoms. The effect size ranges from small–moderate (TMS over the mPFC/ACC and OFC) to moderate (TMS over the pre-SMA and DLPFC). The moderator analyses did not show any clear clinical predictor of post-treatment Y-BOCS score reduction. The current evidence is still not sufficient to provide clear indications of a TMS protocol and/or a brain target over the others. This fact is at least partially due to the small sample size and heterogeneity of the TMS protocols and devices across studies. Thus, future controlled multicenter trials directly comparing different targets and protocols are needed. On the other hand, all the different TMS protocols used across the studies were substantially well tolerated and no serious side effects occurred across all studies, with mild and transient headache as the most frequently reported side effect. In the current literature on OCD, only a single case report exists showing epileptic crisis emerging after TMS (specifically after the first session of the FDA-approved protocol of 20 Hz over the mPFC/ACC) [67]. In addition, in that case, the correlation between TMS treatment and seizure onset is not clear. Therefore, as for other disorders, TMS still confirms in the OCD field its substantial safety and tolerability even with respect to most pharmacological approaches. This latter aspect should be taken into account when considering that the current most evidence-based pharmacological augmentation strategy for resistant OCD is the prescription of antidopaminergic agents (medication with poor metabolic tolerability and which could also affect the epileptic threshold). Several aspects of TMS for OCD remain controversial and substantially open fields to be investigated in future studies. One important future goal will be to achieve TMS protocol optimization in order to maximize the balance of cost/effectiveness for each patient. For this purpose, future studies should define the sufficient number of sessions and stimuli for each patient as well as define clinical features or biomarkers to predict the most promising TMS target for a single patient. In addition, defining strategies to augment the TMS effects should be investigated. In this view, the studies targeting the mPFC/ACC coupled the TMS treatment with a symptom provocation protocol during the stimulation in order to activate the targeted circuitries and increase the TMS effect. This approach has not been tested in studies targeting other targets, and its real advantage has not been investigated in controlled studies (e.g., symptom provocation TMS vs. usual TMS). Finally, only a few studies have investigated the effect of theta burst stimulation (TBS) and accelerated protocols on OCD patients. The use of TBS is of particular interest since it allows clinicians to deliver a greater amount of stimuli in a shorter period of time with respect to a conventional rTMS protocol and to administer more than one session per day. Thus, TBS protocols could become a more affordable and cost-effective treatment approach for treating OCD patients. 

## 5. Conclusions

In conclusion, TMS of several brain targets represents a safe and effective treatment option for OCD patients. However, further studies are needed to help clinicians to define which protocol and which brain target should be considered for each patient.

## Figures and Tables

**Table 1:** Summary of TMS open and sham-controlled trials for OCD.

	Brain Target	Study Design	Stimulation Type/Frequency/Pulse Per Session	Number of Sessions	Subjects (Active Branch)
Mantovani 2010 [12]	Bilateral pre-SMA	Sham-controlled	rTMS/1 Hz/1200	20	9
Gomes 2012 [13]	Bilateral pre-SMA	Sham-controlled	rTMS/1 Hz/1200	10	12
Hawken 2016 [14]	Bilateral pre-SMA	Sham-controlled	rTMS/1 Hz/1800	25	10
Pelissolo 2016 [15]	Bilateral pre-SMA	Sham-controlled	rTMS/1 Hz/1500	20	19
Arumugham 2018 [16]	Bilateral pre-SMA	Sham-controlled	rTMS/1 Hz/1200	18	19
Harika-germaneau 2019 [17]	Bilateral pre-SMA	Sham-controlled	cTBS/600	30	14
Zhang 2019 [18]	Bilateral pre-SMA	Sham-controlled	rTMS/1 Hz/1200	20	25
Mukherjee 2022 [19]	Bilateral pre-SMA	Sham-controlled	cTBS/900	30	13
Guo 2022 [20]	Bilateral pre-SMA	Sham-controlled	cTBS/1200	20	26
Pallanti 2016 [21]	Bilateral pre-SMA	Open label	rTMS/1 Hz/1200	15	25
Donse 2017 [22]	Bilateral pre-SMA	Open label	rTMS/1 Hz/1000	10	22
Mantovani 2021 [23]	Bilateral pre-SMA	Open label	rTMS/1 Hz/3600	10	8
Gajadien 2022 [24]	Bilateral pre-SMA	Open label	rTMS/1 Hz/1200	10	35
Alonso 2001 [25]	rDLPFC	Sham-controlled	rTMS/1/Hz/1200	18	10
Prasko 2006 [26]	lDLPFC	Sham-controlled	rTMS/1 Hz/1800	10	18
Sachdev 2007 [27]	lDLPFC	Sham-controlled	rTMS/10 Hz/1500	10	10
Badawy 2010 [28]	lDLPFC	Sham-controlled	rTMS/20 Hz	15	20
Sarkhel 2010 [29]	rDLPFC	Sham-controlled	rTMS/10 Hz/800	10	21
Mansur 2011 [30]	rDLPFC	Sham-controlled	rTMS/10 Hz/2000	30	13
Ma 2014 [31]	Bilateral DLPFC	Sham-controlled	rTMS/8–12 Hz/648–872	10	25
Haghighi 2015 [32]	Bilateral DLPFC	Sham-controlled	rTMS/20 Hz/750	10	10
Elbeh 2016 [33]	rDLPFC	Sham-controlled	rTMS/1 H or 10 Hz/2000	10	15
Seo 2016 [34]	rDLPFC	Sham-controlled	rTMS/1 Hz/1200	15	14
Shayganfard 2016 [35]	Bilateral DLPFC	Sham-controlled	rTMS/20 Hz/750	10	5
Jahangard 2016 [36]	Bilateral DLPFC	Sham-controlled	rTMS/20 Hz/750	10	5
Naro 2019 [37]	lDPFC	Sham-controlled	iTBS/600	20	5
Khedr 2022 * [38]	rDLPFC	Sham-controlled	rTMS/1 Hz/1500	10	20
Jahanbakhsh 2023 [39]	lDLPFC	Sham-controlled	rTMS/1 Hz/1200	15	15
Williams 2021 [40]	rPFC	Open label	cTBS/1800	50	7
Topcuoglu 2022 [41]	lDLPFC	Open label	rTMS/1 Hz/1200	30	27
Ruffini 2008 [42]	lOFC	Sham-controlled	rTMS/1 Hz/600	15	16
Nauczyciel 2014 [43]	rOFC	Sham-controlled	rTMS/1 Hz/1200	10	10
Dutta 2021 [44]	lOFC	Sham-controlled	cTBS/600	10	18
Liu 2021 [45]	rOFC	Sham-controlled	cTBS/600	20	12
Khedr 2022 [38]	rOFC	Sham-controlled	rTMS/1 Hz/1500	10	20
Dunlop 2016 [46]	Bilateral dmPFC	Open label	rTMS/10 Hz/6000	20/30	20
Carmi 2018 [47]	mPFC/ACC	Sham-controlled	dTMS/20 Hz/2000	25	16
Carmi 2018 * [47]	mPFC/ACC	Sham-controlled	dTMS/1 Hz/900	25	8
Carmi 2019 [48]	mPFC/ACC	Sham-controlled	dTMS/20 Hz/2000	29	42
Roth 2021 [49]	mPFC/ACC	Open label	dTMS/20 Hz/2000	29	185
Reddy 2022 [50]	mPFC/ACC	Open label	dTMS/20 Hz/2000	10	15
Arikan 2022 [51]	mPFC/ACC	Open label	dTMS/20 Hz/2000	30	29
Ikawa 2022 [52]	mPFC/ACC	Open label	dTMS/20 Hz/2000	30	26
Kang 2009 [53]	rDLPFC + bilateral pre-SMA	Sham-controlled	rTMS/1 Hz/1200 + 1200	10	10
Tadayonnejad 2022 [54]	DLPFC + SMA + rOFC	Open label	rTMS/1 Hz/1200 + 1200 + 1200	17 ± 6	18

SMA: supplementary motor area; DLPFC: dorsolateral prefrontal cortex; OFC: orbitofrontal cortex; mPFC: medial prefrontal cortex; dmPFC: dorsomedial prefrontal cortex; ACC: anterior cingulate cortex; rTMS: repetitive transcranial magnetic stimulation; cTBS: continuous theta burst stimulation; iTBS: intermittent theta burst stimulation; * second branch of the study.

## Data Availability

Not applicable.

## Supplementary Materials

The following supporting information can be downloaded at: https://www.mdpi.com/article/10.3390/life13071494/s1. Table S1: PRISMA checklist.
